# The impact of ostomy on colorectal cancer patients and caregivers: a qualitative study

**DOI:** 10.1007/s00520-025-10075-x

**Published:** 2025-11-08

**Authors:** Tseganesh Asefa, Hiwot Tezera Endale, Mihret Getnet, Hailu Aragie, Habtu Kifle Negash, Yibeltal Yismaw Gela, Winta Tesfaye

**Affiliations:** 1 Department of Medical Nursing, School of Nursing, College of Medicine and Health Science, University of Gondar, Gondar, Ethiopia; 2 Department of Medical Biochemistry, School of Medicine, College of Medicine and Health Sciences, University of Gondar, Gondar, Ethiopia; 3 Department of Epidemiology and Biostatistics, Institute of Public Health, College of Medicine and Health Sciences, University of Gondar, Gondar, Ethiopia; 4 Department of Human Anatomy, School of Medicine, College of Medicine and Health Sciences, University of Gondar, Gondar, Ethiopia; 5 Department of Human Physiology, School of Medicine, College of Medicine and Health Sciences, University of Gondar, Gondar, Ethiopia

**Keywords:** Caregivers, Colorectal cancer, Ethiopia, Ostomy, Patients, Qualitative study

## Abstract

**Introduction:**

Colorectal cancer is the third leading cause of cancer-related deaths worldwide. Its incidence has been rising in Africa due to urbanization and changing lifestyles. In Ethiopia, the lack of early diagnosis and specialized care places an additional burden on patients. Ostomy surgery, commonly used to manage advanced cases of colorectal cancer, significantly affects patients’ quality of life. Despite the well-documented challenges, there is a limited number of studies investigating the experiences of Ethiopian colorectal cancer patients and their caregivers. This study aims to explore the impact of living with an ostomy on both patients and their caregivers.

**Methods:**

The qualitative descriptive study with a phenomenological approach was conducted from February to May 2024 at St. Paulo Comprehensive Specialized Hospital, Ethiopia. Purposive sampling was used to recruit adult colorectal cancer patients with ostomies and their caregivers. In-depth individual and shared interviews were conducted using a semi-structured guide. Data quality assurance was maintained and analyzed using thematic analysis guided by family systems theory.

**Results:**

A total of 28 participants (14 patients and 14 caregivers) were included in the study. Thematic analysis identified seven themes across three domains. From the patients’ perspective, three themes emerged: psychological burden (altered self-image and confidence and sense of insecurity), daily challenges (routine care obstacles and lifestyle restrictions), and social detachment (self-stigmatization, self-imposed isolation, and identity loss). Caregivers highlighted two themes: role redefinition (practical challenges and loss of personal freedom) and caregiving burden (work-life balance and financial strain). Additionally, shared interviews with patients and caregivers revealed two further themes: challenges in communication (avoidance of difficult conversations and intimacy dynamics) and boundary negotiation (decision-making tension and evolving responsibilities).

**Conclusion and recommendations:**

The current study described the experience of colorectal cancer patients with ostomies and their caregivers within a family systems theory framework while revealing cultural factors such as social detachment, identity loss, and self-imposed isolation. The findings call for culturally sensitive interventions that address both emotional and social challenges. It emphasizes the need for support systems that encourage social reengagement and open communication, with a focus on holistic care that considers cultural context.

## Introduction

Colorectal cancer is the third leading public health problem; it accounts for around 10% of all diagnoses of cancer throughout the world and is recorded further as the second cause of death from malignant cancer [1, 2]. In 2022, according to an estimate by the Global Cancer Observatory, a total of 1.9 million new colorectal cancer cases occurred and accounted for over 930,000 deaths. These are predicted to rise by 73% in 2040 [3, 4]. The burden of colorectal cancer is higher in high-income countries, which might be attributed to an unhealthy lifestyle among individuals: a diet high in processed meat and low in fruits and vegetables, physical inactivity, a high body mass index, smoking, and a high intake of alcohol are considered risk factors [5–7]. However, studies indicate that through effective screening programs, the incidence of colorectal cancer has decreased in those countries [8]. The colorectal cancer burden is increasing in Africa due to factors such as urbanization, dietary shifts, and rising life expectancy. Similarly, colorectal cancer has emerged as one of the major public health priority concerns in Ethiopia, with estimates of 8 cases per 100,000 person-years [9, 10]. Furthermore, due to a lack of early diagnosis and treatment options, it often presents at advanced stages, which also requires specialized care. However, specialized services and support are not always available, with wide variation in their availability across the country, adding further burdens to patients [11, 12].

Surgical treatments, such as the creation of an ostomy, are relatively common in the management of colorectal cancer, with estimates ranging from 18 to 35% of patients undergoing ostomy procedures during the course of their treatment [13]. While such surgeries are vital for managing cancer and improving survival rates, they alter the lives of patients in many ways, affecting most aspects of physical, emotional, and social functioning [14–16].

Research indicates that a significant percentage of these patients require ostomy surgery, yet there is a lack of comprehensive understanding regarding their postoperative experiences. Patients describe physical difficulties, including challenges from ostomy care, dietary restrictions, and the need for ongoing adaptation to daily routines. The psychological impact is also profound; patients experience anxiety, depression, and concerns about body image and self-esteem. In a societal context where health perceptions and body image are closely intertwined with cultural expectations, these challenges may be magnified, resulting in heightened feelings of social isolation and stigma [17–20].

The experiences of caregivers of colorectal cancer patients are equally crucial yet frequently overlooked. Caregivers face a unique set of challenges, including the emotional burden of witnessing a loved one’s struggles and managing the practical aspects of care [21–23]. This role can lead to feelings of stress, anxiety, and a sense of diminished personal freedom as caregivers balance their responsibilities with their own well-being. In the African context, traditional gender roles and cultural expectations can further complicate these dynamics, placing additional burdens on caregivers who often navigate these societal norms while providing support [24–26].

Despite the increasing recognition of the psychosocial dimensions of living with an ostomy, there remains a notable gap in the literature regarding its specific impact on colorectal cancer patients and their caregivers, particularly in the Ethiopian context. This study aims to explore the impacts of living with an ostomy on colorectal cancer patients and their caregivers, with an emphasis on the physical, emotional, social, and practical dimensions of their experiences.

## Methods and materials

### Study period and setting

The study was conducted from February to May of 2024 at St. Paulo Comprehensive Specialized Hospital. The hospital is a major referral center with oncology units providing treatment and follow-up for different cancer patients, including colorectal cancer.

### Study design

This study employed a descriptive phenomenological approach, as outlined by Husserl and adapted in health research [27–29], which aims to explore the lived experience of colorectal cancer patients living with ostomies and their caregivers’ experiences as they are perceived, without imposing preconceived interpretations.

The study was guided by family systems theory, which views families as interconnected systems where changes in one member’s health can influence the functioning of the entire family unit [30, 31]. Integrating this theory with phenomenology allowed the study to explore both individual experiences and relational dynamics. While phenomenology focused on participants’ personal experiences of living with an ostomy, family systems theory provided a complementary lens to understand how these experiences shaped—and were shaped by—interactions, roles, and communication patterns within the family. This integration ensured philosophical coherence, maintaining the descriptive phenomenological emphasis on participants’ perspectives while highlighting relational processes relevant to caregiving.

### Study population

The study involved colorectal cancer patients aged 18 years and above who underwent ostomy surgery more than three months ago and are on follow-up care at St. Paulo Comprehensive Specialized Hospital. Caregiver partners (spouses, family members, or significant others) aged 18 years and above were included if they were directly involved in the patient’s care. The patient selected the caregiver partner based on their primary caregiving role. Individuals with cognitive or communication impairments were excluded.

### Sample size determination and sampling technique

A purposive sampling strategy was used to recruit participants who could provide rich and detailed information about the lived experiences of living with an ostomy [32, 33]. The total sample size was 28, comprising 14 patients and their 14 caregiver partners. After each interview, data were reviewed to identify emerging insights, and recruitment continued until data saturation was reached, defined as the point at which no new themes or significant information emerged from additional interviews.

### Data collection tool and technique

**Fig. 1:**
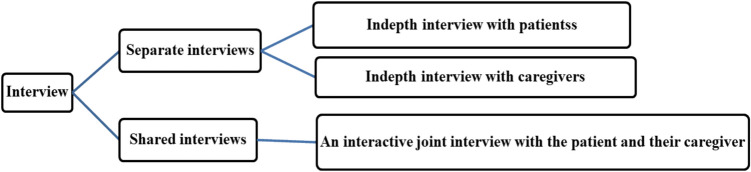
Interview structure for cancer patients and caregivers

An interview guide was initially prepared in English through the critical review of the study objectives and relevant literature. It was then translated into Amharic by a fluent bilingual speaker to maintain linguistic and contextual accuracy. Data collection was conducted in two stages: (1) individual in-depth interviews with patients and caregiver partners separately to explore personal experiences and challenges; and (2) an interactive shared interview with patients and their caregiver partners together, designed to capture relational dynamics, shared coping processes, and interactional patterns (Fig. 1).

Potential patient participants were recruited by oncology nurses. Patients who consented to participate were then invited to nominate a primary caregiver to take part in the study. Both patients and their selected caregivers were informed about the study purpose, the ethical approval obtained, and the interviewer’s credentials. Written informed consent to participate in this study was obtained from all participants before conducting the interviews, which included consent for publication of this study. The individual separate interview preceded shared interviews; interviews were conducted face-to-face in a private setting within the hospital to maintain confidentiality and facilitate open discussion. These interviews discussed the emotional and psychological impacts of living with an ostomy, changes in social relationships, and practical difficulties regarding physical care and support systems. Individual interviews lasted between 30 and 40 min, and shared interviews were taken to 35 min to avoid participant fatigue. All documents were securely archived, and digital material was password-protected; only the research team was granted access to maintain participant confidentiality and protect the integrity of the study.

### Data processing and analysis

Before analysis, interviews’ field notes and audio recordings were transcribed and then translated from Amharic to English verbatim by a fluent speaker of both languages. The researcher then entered and saved the data as Microsoft Word documents for analysis. Data were analyzed thematically following the six-step framework of Braun and Clarke (2006) [34], which includes (1) familiarization with data, (2) generating initial codes, (3) searching for themes, (4) reviewing themes, (5) defining and naming themes, and (6) writing the report. First, familiarization with data occurred through transcript reading and rereading. Next, initial codes were generated separately for patients, caregivers, and shared experiences. Then, related codes were organized into subthemes and broader themes. After that, themes were reviewed and refined for accuracy and coherence. Finally, findings were written up with illustrative participant quotations, ensuring that the analysis remained grounded in participants’ lived experiences.

Qualitative data analysis software, such as OpenCode, was employed to ensure that the data were systematically organized and interpreted. Quotes from participants support this finding, with only minor adjustments in grammar and linguistics made in order to increase clarity without changing the meaning. This was done as a means of ensuring the analysis remains within participants’ voices and serves effectively to highlight key insights emerging from the data.

### Data quality assurance

To ensure the trustworthiness of this qualitative study, several strategies were employed to address credibility, dependability, confirmability, and transferability [35]. Credibility was attained through data source triangulation by relying on both individual and shared caregiver interviews for capturing an in-depth representation of experiences. In this respect, dependability was achieved by a thorough description of the research process, including the use of a consistent interview guide, thematic analysis, and documentation of changes of any kind during the time of research.

Confirmability was achieved by minimizing the bias of the researcher, as the iterative analysis was supported by audio recordings and verbatim transcriptions. The qualitative software used was for systematic data management in order to ensure that findings are themselves driven by data rather than being based on personal assumptions. This helped to reinforce the objectivity and trustworthiness of the data. Maximum variability had been attained in achieving transferability through the rich, thick description of the context of the study, participants, and setting, which allows other researchers or practitioners to identify the relevance of findings to similar settings or populations.

## Results

### Sociodemographic and clinical characteristics of the study participants

In the study, the majority of patients with colorectal cancer were between the ages of 46–60 years (42.9%) and above 61 + years (35.7%), while 57.1% were males. In terms of the level of education, 28.6% were educated up to the primary school level, while another equal number had completed secondary school. Regarding employment status, 35.7% were employed, while another 35.7% were retired. Of patients, currently, 71.4% were married or partnered, and 64.3% had an advanced stage of cancer (III-IV). Half of them had ostomy surgery within 6 months before, and 71.4% had a colostomy. Also, 64.3% of patients had medical insurance (Table 1).

**Table 1:** Socio-demographic and clinical characteristics of colorectal cancer patients (*n* = 14)

Characteristic	Category	Frequency (%)
**Age (years)**	18–30	1 (7.1%)
	31–45	2 (14.3%)
	46–60	6 (42.9%)
	61 +	5 (35.7%)
**Gender**	Male	8 (57.1%)
	Female	6 (42.9%)
**Educational level**	No formal education	3 (21.4%)
	Primary school	4 (28.6%)
	Secondary school	4 (28.6%)
	Higher education	3 (21.4%)
**Employment status**	Employed	5 (35.7%)
	Unemployed	4 (28.6%)
	Retired	5 (35.7%)
**Marital status**	Married/Partnered	10 (71.4%)
	Single	2 (14.3%)
	Divorced/Widowed	2 (14.3%)
**Stage of cancer**	Early-stage (I-II)	5 (35.7%)
	Advanced-stage (11-IV)	9 (64.3%)
**Time since ostomy surgery**	3–6 months	7 (50.0%)
	7–12 months	5 (35.7%)
	1 + year	2 (14.3%)
**Type of ostomy**	Colostomy	10 (71.4%)
	Ileostomy	4 (28.6%)
**Medical insurance**	Yes	9 (64.3%)
	No	5 (35.7%)

Among the caregivers, 35.7% were aged 46–60 years, and 57.1% were female. Most of the caregivers (64.3%) resided in urban areas. Half of the caregivers (50.0%) were employed, and 71.4% did not have any health problems. The majority of caregivers (57.1%) were the spouse of the survivor, and 71.4% lived with the survivor. Furthermore, 57.1% of the patients had one caregiver assisting them (Table 2).

**Table 2:** Socio-demographic characteristics of caregivers (*n* = 14)

Characteristic	Category	Frequency (%)
**Age (years)**	18–30	2 (14.3%)
	31–45	3 (21.4%)
	46–60	5 (35.7%)
	61 +	4 (28.6%)
**Gender**	Male	6 (42.9%)
	Female	8 (57.1%)
**Place of residence**	Urban	9 (64.3%)
	Rural	5 (35.7%)
**Employment status**	Employed	7 (50.0%)
	Unemployed	4 (28.6%)
	Retired	3 (21.4%)
**Health problems**	Yes	4 (28.6%)
	No	10 (71.4%)
**Relation with patients**	Spouse	8 (57.1%)
	Child	3 (21.4%)
	Sibling	2 (14.3%)
	Other	1 (7.1%)
**Living with a patient**	Yes	10 (71.4%)
	No	4 (28.6%)
**Number of assisted caregivers**	1	8 (57.1%)
	2	4 (28.6%)
	3 or more	2 (14.3%)

### The impact of ostomy on colorectal cancer patients and their caregivers

**Fig. 2:**
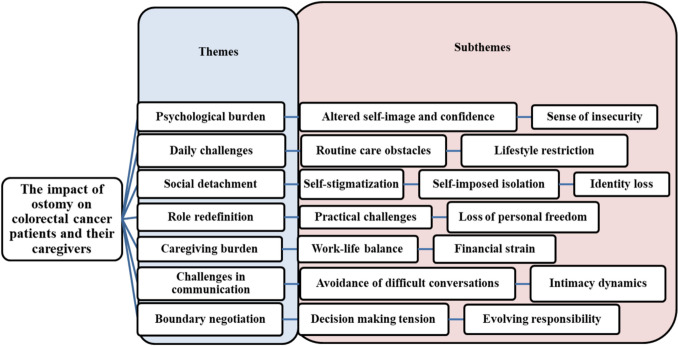
Overview of themes and subthemes related to the impact of ostomy on colorectal cancer patients and their caregivers

The study reveals seven significant themes drawn from comprehensive interviews with colorectal cancer patients and their caregivers. The first three themes emerge from separate in-depth interviews with patients, addressing the “psychological burden,” “daily challenges,” and “social detachment” encountered in their journeys. Themes 4 and 5 arise from separate in-depth interviews with caregivers, focusing on “role redefinition” and “caregiving burden.” Themes 6 and 7 result from a shared interactive interview with patients and their caregivers; the results are “challenges in communication” and “boundary negotiation” (Fig. 2).

#### Theme 1: Psychological burden (patients’ in-depth interviews)

Ostomy imposes a strong psychological burden on colorectal cancer patients, which leads to a variety of emotional problems such as anxiety, vulnerability, and alteration of self-identity. Many patients continued to experience ongoing problems related to body image, which resulted in low satisfaction and reduced self-esteem. Survivor participant 5 expressed the effects of altered body image, saying, “*I look in the mirror and don’t recognize myself... The emotional weight of living with this bag is heavier than I expected*.” This reflection underscores the deep emotional challenge and identity struggle faced by individuals adapting to life with an altered body image due to an ostomy.

The constant fear connected with these issues advances feelings of shame and discomfort, which might cause them to avoid social situations. All this pressure is constantly undermining self-confidence, interfering with daily functioning, and negatively influencing mental health. It consisted of two subthemes: “altered self-image and confidence” and “sense of insecurity”; these respectively point out different facets of the psychological complications related to living with an ostomy.

##### Altered self-image and confidence

Living with an ostomy has significantly impacted patients’ self-confidence, leading many to feel that their self-esteem has diminished. This loss of confidence stems from the challenges of adapting to changes in body image, self-acceptance, and social relationships, causing patients to feel less worthy. Survivor participant 8 shared the following: “*Since the surgery, I have lost all confidence in myself. The ostomy feels like a burden, and I can’t stand to look at my body. I don’t feel like the person I used to be, and accepting this new reality has been incredibly difficult*.” Being able to adapt to life after surgery also means finding one’s place among new difficulties that amplify one’s fears. Survivor participant 10 shared, “*Facing new challenges after my surgery intensified my self-doubt. Each obstacle made me question my abilities and left me feeling less confident than before*.” It is not just a lack of confidence in their body, but it extends into how patients view themselves and their role within society. Survivor participant 14 shared, “*I used to be confident in how I looked, but now I feel insecure all the time. The ostomy makes me feel unattractive, and I am constantly worried people will notice it*.” The loss of self-confidence is a very complex problem involving body image, identity, and social dynamics, and calls attention to the emotional struggle associated with living with an ostomy.

##### Sense of insecurity

An important component of the psychological trauma among patients of colorectal cancer with ostomies is a deep-seated feeling of insecurity. This is due to a general fear that the failure of equipment, leakage, odors, or related circumstances can lead to public embarrassment. As survivor participant 12 explained, “*I am never able to relax completely because there is always that fear in the back of my mind—what if the bag leaks? I have to plan everything with such care, and even then, there is no guarantee. It just makes me feel like I am always walking on eggshells*.” This is demonstrated by the way patients feel they must go to extreme measures, such as packing extra goods each time they leave the house. Survivor participant 13 elaborated, “*Every time I go out, I carry a bag full of supplies—extra pouches, wipes, even a change of clothes. The number of things that could go wrong weighs heavily on my mind. The constant fear that at any moment something might occur inhibits me from the enjoyment of anything*.” These ongoing fears create an insistent sense of vulnerability that affects their quality of life, never living with a notion of normalcy or knowing how to relax deeply.

#### Theme 2: Daily challenges (patients’ in-depth interviews)

Patients with colorectal cancer have to live with considerable daily challenges related to having an ostomy, which influences their quality of life. Activities once considered routine—personal hygiene, selection of clothes, and meal planning—now require pre-planning and accommodation. Constant attention to the ostomy bag, along with worries about potential leaks or discomfort, makes even simple tasks feel daunting. Survivor participant 7 explained, “*Living with an ostomy has turned my daily life upside down. I spend so much time managing my care, and it feels overwhelming at times.*” This reflection captures the emotional toll of the daily management required. This was reflected by another participant when survivor participant 4 shared, “*Every day is a challenge. I have to think twice before I do anything—what I wear, where I go, even what I eat. It feels like my whole life revolves around managing this bag*.” This verbatim further illustrates great adjustments and continuing struggle in conducting life with an ostomy. The theme has two subthemes: “routine care obstacles” and “lifestyle restrictions.”

##### Routine care obstacles

Routine care often disrupts daily life, requiring ongoing attention to tasks like managing ostomy supplies, maintaining skin integrity, and preventing leaks. Survivor participant 2 said, “*Every day is a challenge… I worry that I do everything right and find it very exhausting*.” Another participant explained the stress of leakage. Survivor participant 9 said, “*I have been having so much trouble with leaks that I feel that I am in this evil circle. Sometimes I develop irritation and even wounds since I am constantly trying to find ways out of the leaks, which is stressful and painful*.”

Additionally, patients have to deal with issues other than the physical care of the stoma. Many have to make changes in clothing, as most find their old clothes are no longer comfortable or cannot cover up the ostomy bag as required, thus necessitating new purchases to meet their needs. As survivor participant 5 expressed, “*Before, I used to wear clothes appropriate for the weather; you know that it is hot most of the year in Ethiopia. But now, I wear a jacket throughout the year in order not to show my bag. I lost my most suitable shirts that I used to wear before*.” Survivor participant 6 further explained, “*I can’t wear my favorite outfits anymore because they don’t fit well with the bag, and I have to be very careful about what I eat to manage gas. It feels like I am constantly on high alert.*” Also, dietary limitations become a point of concern as patients learn which foods are safe for them and will not result in uncomfortable symptoms or complications, thereby limiting what they can enjoy eating. Survivor participant 8 reported, “*I love beans and broccoli. Unfortunately, I have to avoid those foods all the time because they really make me gassy*.” These routine care challenges extend beyond physical maintenance, affecting various aspects of daily life and overall quality of life for those living with an ostomy. The adjustments add to daily burdens, evoking frustration and a deep sense of loss over the freedoms they once had.

##### Lifestyle restriction

Patients with colorectal cancer who have undergone ostomies have to face considerable changes in lifestyle and have to accommodate themselves to such conditions. This is presenting modifications in daily habits when one engages in diverse activities to create room for health needs. The changes touch on most aspects of their life: work, sociability, and even exercise. As survivor participant 1 explained, “*I have been in the business of cooking for about ten plus years now. I do and always did like to prepare food. My health problems have made it very painful to stand in a hot kitchen for long. Now I work with preparing street food, striving to mold my cooking according to my needs for health.*” This verbatim reflects not only his professional journey but also highlights the struggle of balancing his passion for cooking with the limitations imposed by his illness.

According to survivor participant 9, participating in physical leisure activities, including exercise, involves careful and detailed planning. “*I enjoy hiking in the mountains, but being away from home makes it stressful. I worry about my bag rupturing, running out of space, and figuring out how to manage my ostomy when it needs to be emptied.*” This shows the common changes patients must adapt to in order to continue comfortably while living with an ostomy. These types of life adjustments show that living with an ostomy has extensive impacts on patients’ daily lives, which include professional, social, and recreational living. The constant struggle of having to adjust to and address their own health challenges exemplifies the resiliency required in living with an ostomy, while at the same time offering insight into the emotional and practical challenges they are faced with.

#### Theme 3: Social detachment (patients’ in-depth interviews)

Social detachment reflects feelings of isolation and disengagement from relations with others, despite social interaction, as colorectal cancer patients with ostomies commonly experience. Indeed, the physical and emotional difficulties of living with an ostomy tend to induce in them a profound sense of separation from friends, family, and community that heavily impacts their quality of life. Many patients are apprehensive about social activities due to the fear of being judged or embarrassed about their ostomy. Indeed, feelings of difference often lead to withdrawal from social situations where participants voice concern about how others may perceive them.

Patient participant 3 shared, “*In the past, going out with friends for dinner, dancing, and having the time of one’s life. Now these things bring out anxiety. I am always thinking about how to manage my ostomy after eating, which makes me strain or not enjoy their company. Hence, I am mostly not joining them*.” This highlights the psychological burden that the condition could inflict on socializing. Another survivor participant, 11, explained, “*Since the ostomy, I have been losing a piece of myself. I avoid going places for fear of being judged and causing an accident, which results in embarrassment, and I don’t connect to the traditional ceremonies anymore. It is easier to be in isolation, but that leaves me disconnected and as if a part of me has lost its identity*.” Where there is a feeling of judgment and embarrassment with loss of identity, it generally leads to a retreat from people, further contributing to isolation. This theme was categorized into three subthemes: “self-stigmatization,” “self-imposed isolation,” and “identity loss.”

##### Self-stigmatization

Self-stigma is one of the big issues for colorectal cancer patients who have to live with an ostomy; the fact that individuals internalize the stigma set forth by society may lead to feelings of lowliness, shame, and self-inferiority. Generally, these negative perceptions emanate from misconceptions and cultural expectations of living with an ostomy. This makes patients retract into their comfort zones to avoid perceived judgments and embarrassment, alienating them further and worsening this feeling of solitude. The shame may impede their interpersonal interactions in society.

In the words of survivor participant 6: “*I feel like I lost my worth because of the ostomy; I lost my value. I often stay home because I am afraid of what people will think of me*.” Another survivor participant, 9, shared, “*I feel as though my body betrayed me. There is this bag; it’s always there to remind me that I am not whole anymore. I always took great pride in how I presented myself, but now I am unable to even stand looking at myself. Hard to imagine anyone else could either.*” These reflections give a sense of how internalized stigma influences patients socially and leads them to retreat from meaningful social contact, ultimately affecting their emotional lives.

##### Self-imposed isolation

The most shared coping strategy among patients with colorectal cancer who had an ostomy was the fear of complications from ostomy management, which led them to withdraw from social activities and social relations. This constant threat of possible leakage, searching for an adequate bathroom, or even the chance that others might notice it brings about in patients a preference to isolate themselves from social interactions rather than be exposed to the perceived risks and stresses.

Survivor participant 7 shared, “*I avoid going out because I am always anxious about something going wrong with the bag. What if it leaks, or I am unable to find a bathroom in time? It’s just easier to stay home and avoid the stress.*” The fear of losing control in public settings may lead patients to make radical changes to daily routines, such as eating alone rather than with colleagues. Whereas earlier, “*I used to enjoy lunch breaks with my colleagues; now I eat alone in my office*.” This survivor participant 10 explained and expressed a movement from social experiences to an enclosed one.

The need to avoid situations felt to be uncontrollable can add stress to friendships and other social relationships as well. Survivor participant 4 said, reflecting on how hard it can be to keep social contacts, “*My friends always invite me out, but I have started making excuses. I don’t feel comfortable going to places where I can’t control the situation*.” At the same time, concern over what others may think about the ostomy influences patients’ choices of physical activities. Survivor participant 14 explained the following: “*I stopped going to the gym because I was afraid people might notice the ostomies or I would have to explain why I can’t do certain exercises anymore*.” It is from these reflections that it is clearly shown this concern with the management of an ostomy creates a spiral of self-imposed isolation that has consequences for patients’ social interaction and overall well-being.

##### Identity loss

Patients of colorectal cancer with an ostomy usually face a big challenge in trying to reconcile their self-concept in view of the new physiological status, finding coherence with the past self. An ostomy often creates a profound difference in one’s self-concept and identity when related to roles and continuance with traditional ceremonies, hobbies, or physical activities. Individuals feel disconnected from activities that defined them. This can cause them to feel disconnected from an important part of their identity and reduce their feelings of belonging.

Patient participant 2 explained, “*Sharing food and drinks with my family used to be a thing that brought joy, but now I am embarrassed. It makes me very wary of joining in, and I usually feel left out.*” Another survivor, participant 12, mentioned, “*I am always apprehensive to participate in any cultural meals and dances, since it makes me feel exposed, as if my worth is somehow linked to my ostomy*.” These highlight the daily challenges patients face in trying to navigate their changed identity, which more often than not will lead one to experience oneself as an outsider within one’s own social and cultural orbit.

#### Theme 4: Role redefinition (caregivers’ in-depth interviews)

Caregivers must adapt to new roles involving physical, emotional, and psychological support, often feeling overwhelmed by these evolving dynamics. Balancing caregiving duties with personal needs can create emotional stress, as reflected by caregiver participant 4: “*I want to be supportive, but it’s hard to keep my own feelings in check while managing everything for my partner*.” This declaration stresses the complexities of role redefinition, highlighting the emotional toll of balancing caregiving with personal needs. This theme was further categorized into “practical challenges” and “loss of personal freedom.”

##### Practical challenges

Caregivers of colorectal cancer patients with an ostomy have to face routine challenges on a daily basis, such as physical tasks including changing ostomy bags, cleaning stoma sites, and ensuring adherence to dietary and hygiene practices. Many times, caregivers often feel ill-equipped to do such tasks and therefore, anxious and stressed. Indeed, caregiver participant 7 stated, “*I had no idea how to handle an ostomy bag. The first weeks were overwhelming because I was always frightened not to do anything wrong.*” Besides these physical demands, the caregivers have to deal with practical problems like washing of clothes and sheets when leakage results in their soiling. This extra work can become physically exhausting and emotionally daunting. Caregiver participant 9 recalled this challenge: “*After dealing with leaks, I often feel weak and exhausted. The washing of the dirty clothes is endless, and it’s hard to keep up with everything.*”

Caregivers also have to struggle through the arrangement of medical appointments and a balance between personal and professional life. Caregiver participant 10 said, “*It’s tiring to manage between work, home, and caregiving. I get up in the middle of the night and change the ostomy bag, and then I have to wake up early for work in the morning*.” These practical challenges underpin that caregiving is something very complex and long-lasting in nature, which calls very much for the realization of more support and resources.

##### Loss of personal freedom

Loss of personal freedom can be interpreted as the feeling of restraint developed in caregivers because the demands of caregiving seem to inhibit freestyle living. Many caregivers find themselves unable to pursue personal interests or socialize, let alone take time to themselves, since their responsibilities for the survivor’s ostomy-related needs have started to consume them. This is a loss of freedom, part of their quality of life, which, while attempting to balance caregiving duties with personal desires and needs, may make one feel isolated and frustrated. As caregiver participant 1 said, “*I used to go out with friends or have time for myself, but now it’s as if all my life is taken up by caring for him. It feels like I have lost who I was.*” Caregiver participant 8 testified, “*I used to love going for morning walks, but now it feels like every moment is dedicated to taking care of his needs. It does feel that there is no time left on my hands for myself anymore*.”

#### Theme 5: Caregiving burden (caregivers’ in-depth interviews)

Caregiving burden describes the deeper emotional, physical, and logistical stress that caregivers of colorectal cancer patients with ostomies face. Many times, this leads to overwhelming stress and exhaustion in attempting to balance these dual roles of supporting loved ones while managing personal life. Caregivers are often caught between multiple fronts that leave them with feelings of inadequacy and emotional burnout. In this process, caregivers sacrifice their needs for those of the survivor and may suffer health problems along with a decrease in quality of life. Caregiving strains relationships and may become a spiral of anxiety as each tries to balance the needs for health and comfort of the partner while hanging onto their identity and lifestyle.

This is what caregiver participant 11 reported: “*Every day is a struggle. I want to be there for my husband, but at the same time, I feel like I am losing out on myself. Sometimes, I don’t even have the time to eat properly or sleep. It’s exhausting*.” The reflection one sees here is a sacrifice that the person is making by being a caregiver; it explains not only that the demands of caregiving are incessant but also that a lot of times they crush an individual’s sense of identity. It involves “work-life balance” and “financial strain” as the subthemes of this theme.

##### Work-life balance

The demands of caregiving cause severe work-life balance problems because the responsibility has to shift further and further to caregiving. The demands of caregiving interfere with caregivers’ professional duties, leading to absent workdays or hours and ways of lowering productivity at work. Most of the caregivers feel guilty if they cannot allocate ample time to their job or when they have to leave the office for medical appointments or emergencies. This may breed great stress, as the caregivers feel they need to perform well in the two roles, thereby pitting work responsibilities and caregiving duties against each other continuously. Further tension produced by managing work and caregiving responsibilities often creates burnout, where feelings of being trapped and overwhelmed tend to be commonplace for the caregivers.

Caregiver participant 2 explained, “*I had to take a leave of absence from work to care for my wife. It is hard to explain to my boss why I need to be away so much*. *I fear that my job security is in jeopardy due to my caregiving responsibilities.*” Another caregiver participant, 6, said, “*I run a mini shop that supplements our income on a monthly basis, and every time I need to go to the hospital, it needs to be closed. When I am home taking care of my partner, it is also closed. We have much lower incomes than we used to have over time.*” Therefore, these statements reflect how emotionally scarring the experience of juggling work and caregiving really is, with fears of losing one’s job and relentless pressure to perform in both spheres.

##### Financial strain

The financial consequences for caregivers further complicate caregiving. Medical care, supplies for ostomy, potential loss of income due to reduced work hours—the list goes on and on and adds up to some fairly substantial burdens. Thus, it puts pressure on the caregivers to handle such expenses, causing anxiety over economic stability. The major concern of the majority of the caregivers was keeping the household finances in place while trying to provide enough care to their loved ones. This can therefore create some level of financial burden for the caregivers, who would rather seek services that might have been deemed necessary. This is usually a circular effect; hence, it affects their emotional states and consequently the quality of care provided.

As caregiver participant 9 identified, “*The money I spend on supplies is draining our savings. I just can’t afford enough ostomy bags for her, so I’ve been trying to make each one last as long as possible. Sometimes I have to wash it, and she reuses it again and again*.” Another caregiver participant, 14, added, “*I didn’t expect how fast the costs would add up. I am anxious every month over her medicines and ostomy supplies. I frequently have to skip meals just so we can have enough for her needs*.” These are the thoughts that indicate how hard reality can be for many caregivers in trying to manage the financial burdens that come with caregiving, which all too often requires placing loved ones’ needs ahead of basic needs.

#### Theme 6: Challenges in communication (a shared interactive interview with patients and their caregivers)

In their mutual dependence, patients and caregivers draw strength from one another; however, talking about these issues openly with each other is a challenging task. The most delicate ones—connected with body image, sexual closeness, and caregiving stress—become taboo because of their mutual concerns about not wanting to burden each other. This leads to misunderstandings and emotional distance. Survivor participant 5 shared, “*I know my partner is already doing so much for me, and I don’t want to make things harder by talking about how I am really feeling. So I just keep it to myself*.” The struggle was reiterated by a caregiver: “*I see the pain she is in, and I don’t want to add to it. There are days when I am exhausted, but I don’t bring it up because I don’t want her to feel guilty*.” What these sounds really demonstrate is a desire to protect each other, but this very avoidance prevents both parties from articulating their feelings and getting the support they need from each other. This theme then categorized the “avoidance of difficult conversations” and “intimacy dynamics.”

##### Avoidance of difficult conversations

The reasons for avoiding sensitive topic discussions in relation to ostomy living are modification in body structure, caregiving stress, and emotional changes. This is attributed to a feeling of protection from further stress against each other. This may entail misunderstanding and emotional distance. As survivor participant 3 related, “*I know that my partner would like to discuss how my body has changed, yet I catch the hesitation in his eyes. We want to bring it up, but we just don’t*.” This conveys a sense of wanting to communicate and fearing the discomfort of their partner. A caregiver similarly shared, “*I often feel overwhelmed by the care I provide, but I don’t want to burden my partner. It results in superficial conversations, avoiding the deeper issues*.” The effect of this reluctance is isolation and confinement to dialogue, inhibiting either party from professing his needs, with emotional closeness barred.

##### Intimacy dynamics

Colorectal cancer patients and their caregivers are often confronted with the most significant barriers in the discussion of intimacy, even regarding sexual relations after ostomy surgery. Physical changes resulting from such surgery, along with psychological challenges, create an environment that complicates these imperatively necessary conversations. Many caregivers find themselves in turmoil between wanting intimacy and respecting the vulnerability of the other. Caregiver participant 5 reiterated, “*We haven’t spoken much about being intimate since the surgery. It feels sensitive, and I don’t want them feeling uncomfortable*.” On the other hand, survivor participant 2 summarized, “*I just don’t feel the same about my body anymore, and I know it’s impacted our intimacy, but I’m kind of too embarrassed to speak with them about this*.”

The source of this avoidance is wrapped up in concerns about vulnerability and not wanting to hurt one another’s feelings, which work to hinder their adjustment processes relative to the ostomy. Quite commonly, caregivers are uncertain as to how to bring about intimate times without being sensitive to their partner’s insecurities. As Caregiver 7 explained, “*It’s hard to know how to be intimate now. I want to be intimate with my partner, but I see how self-conscious they are with their ostomy. I find myself holding back on numerous occasions, as I don’t wish to make them feel uncomfortable*.”

These feelings expose a very important fact: it is not easy for patients or caregivers to share intimacy and closeness. They live in a world of feelings that are difficult to handle and needs that are hardly ever verbalized, precipitating the feeling of isolation and misunderstanding between the survivor and the caregiver.

#### Theme 7: Boundary negotiation (shared interviews between patients and caregivers)

Boundary negotiation involves the difficulties in balancing personal needs within caregiving demands by caregivers and colorectal cancer patients with an ostomy. Changes brought about by the ostomy test the ability of both parties to understand and redefine roles, establishing new boundaries that take into consideration caregiving demands while striving to protect autonomy and personal well-being. As described by caregiver participant 4, “*Sometimes it’s hard to know where my role as a partner ends and where my role as a caregiver begins. I want to give him the space to do things on his own, but I also feel like I need to be there all the time to make sure everything is okay. It’s exhausting trying to find that balance.*” And survivor participant 3, “*I know she wants to help, but there are moments when I just want to do things myself, even if it takes longer or is harder. It’s frustrating to feel like I have to depend on someone for everything, and sometimes I just need her to let me try on my own.*”

##### Decision-making tension

The challenges that both caregivers and patients face when making care-related decisions. Patients may wish to maintain control over decisions concerning their ostomy care, striving for independence and autonomy. Meanwhile, caregivers may feel obligated to intervene in order to ensure the survivor’s safety and well-being. This tension can lead to conflicts and misunderstandings, affecting the relationship dynamics and creating stress as both parties navigate the delicate balance of decision-making authority.

A survivor participant 1 expressed, “*I want to be the one deciding how I take care of my ostomy, but sometimes she steps in, and it feels like I don’t have a say in my own life.*” A caregiver participant 9 reflected, “*I know he wants to be in control, but I worry about things going wrong if he doesn’t do it right. It’s hard to step back when I just want to keep him safe*.”

As survivor participant 7 explained: “*Sometimes I would just want to decide what I eat or how I manage the bag without anyone questioning me. It is very difficult to feel independent while every decision seems to be a shared decision.*” Another caregiver participant, 14, reflected, “*I understand that he wants to do many things in his own way, but when I watch him struggle, I feel I need to step in. The line between helping and stepping over the edge is a very fine line indeed*.”

##### Evolving responsibilities

The roles of caregivers for colorectal cancer patients with an ostomy evolve as these patients go through a recovery and adaptation process. At first, the caregivers focus on hands-on activities: changing the ostomy bag, helping them clean themselves, and managing daily routines. In time, as patients become more independent, caregivers transition into support roles: emotional support, facilitating lifestyle adjustments, and encouraging independence.’

As caregiver participant 13 explained, “*At first, I felt like I was doing everything—changing the bags, showering them, and even dressing them. Smothering, maybe, but I just wanted to make sure they were comfortable*.” The dynamic here involves a need for the caregivers to back off while still remaining approachable. Patients also have to engage in a complex process of regaining their independence. According to survivor participant 12, “*At the beginning, I relied on the presence of my caregiver, which was hard for me to confront in myself. Nowadays, with time, I do more and more things by myself, and that is good because I take some control back*.” Another survivor participant, 1, put it this way: “*I appreciate everyone’s help, but sometimes I need to do things myself in order to feel normal again*.”

A caregiver participant 12 described, “*One moment, I’m assisting with basic care, and the next, I’m managing medications and scheduling doctor’s appointments. My role has changed so much; it’s like I’m juggling different responsibilities every day*.” Patients likewise suffer as they establish their autonomy. Survivor participant 11 related, “*Having my caregiver do so much for me is humbling. I want to be independent, but I know it’s hard for them to step back. We’re both learning how to navigate this new normal*.” These experiences will reflect some evolving role adjustments that have to be made by both the caregivers and the patients within the emotional and practical complexities.

## Discussion

This study explored the complex and interdependent experiences of colorectal cancer patients with ostomies and their caregivers, revealing psychological, physical, and social challenges. The findings mostly align with family systems theory, which emphasizes the interdependence of family members and how changes in one family member’s health can impact the entire family system. This study also explores unique findings beyond what family systems theory can explain, especially those related to the Ethiopian cultural context and patients’ coping strategies.

Colorectal cancer patients with ostomies often face substantial psychological challenges, including altered self-image and increased insecurity. These issues are compounded by cultural stigmas related to body image, leading to heightened feelings of shame and social withdrawal. The fear of equipment failure and public embarrassment further exacerbates their vulnerability and diminishes their quality of life. These findings are consistent with other research highlighting the psychological impact of ostomy on patients’ quality of life [36–41]. Family systems theory also explains how such emotional challenges extend beyond the individual to the caregiver’s emotional well-being and the overall family dynamic [30].

The other finding of this study is that daily challenges, such as managing ostomy care, dietary adjustments, lifestyle restrictions, and activities like meal planning, clothing choices, and hygiene, require careful planning, leading to emotional exhaustion and a disrupted sense of normalcy. These challenges are well documented in the literature [42–45], emphasizing the significant strain on daily life.

One of the notable findings of this study is the experience of social detachment, characterized by self-stigmatization, self-imposed isolation, and identity loss. Patients often withdraw from social and cultural activities due to fear of judgment or embarrassment, leading to a loss of identity and exacerbated feelings of isolation. This detachment is particularly impactful in cultures where communal interactions are integral to one’s identity. The link between stigma and social isolation among colorectal cancer patients with stomas has been explored in recent studies [46–49]. However, family systems theory fails to explain the disruption in family social roles and the cultural losses tied to preserving identities. Patients with ostomies experience isolation and identity loss due to their inability to participate in culturally significant activities, a topic not widely covered in existing theories. This underscores the need to consider cultural context when studying the impact of illness on family and social dynamics.

Furthermore, patients avoided social interactions and found comfort in the control over their environment to avoid embarrassment and stigma associated with their ostomy. This again contrasts with the usual view of isolation as being bad; instead, self-imposed isolation was a protective mechanism for patients in trying to navigate their new realities. This finding adds to our knowledge on isolation in chronic illness and points to a context in which isolation is both a problematic and an adaptive coping strategy.

The present study highlighted that caregivers frequently assume new responsibilities without adequate preparation or support, leading to emotional and physical exhaustion. Practical challenges, such as managing ostomy equipment and financial strains, further burden caregivers. These findings align with studies on the role conflict experienced by caregivers [50–53], which echoed caregivers taking on new roles and the family having to adjust to accommodate the increased dependency of the survivor. The theory emphasizes how caregiving responsibilities can alter family dynamics, making the caregiver central to the survivor’s daily life and emotional well-being. This adaptation, while necessary, often leads to emotional and physical exhaustion, further supported by the financial burdens identified in this study and existing literature [30, 54].

Another significant finding is that communication barriers between patients and caregivers, especially regarding sensitive topics like intimacy and body image, were evident. Patients often avoided discussing their emotional and physical needs to prevent burdening their caregivers, leading to emotional distance. This emphasizes the need for open communication in the management of chronic illness. Studies [55–57] have shown that avoiding discussions of sensitive topics can lead to emotional distance, unresolved emotional strain, and a lack of intimate communication in cancer care settings. The challenges of discussing intimacy extend beyond what is typically covered by family systems theory. While general communication is addressed, the theory does not explore the emotional avoidance around body image and sexual relationships highlighted in this study. Both patients and caregivers tended to avoid these sensitive topics, leading to emotional distance. This suggests that more focused support is needed to encourage open conversations about intimacy, which could help reduce the emotional strain for both patients and caregivers.

Additionally, the struggle to balance caregiving responsibilities with personal needs resulted in tension, as caregivers felt overwhelmed and patients strived to maintain independence. This dynamic highlights the need for a nuanced understanding of caregiving dynamics, particularly in cultures with strong familial obligations. This finding is consistent with previous studies [58–60].

## Limitations

Although this study gives great insights, it also has limitations that need to be considered. First, reliance on self-reported interviews may have introduced response bias, as participants did not disclose sensitive issues such as body image and intimacy, possibly leading to underreporting.

Besides, this is not a study of longitudinal follow-up, so one cannot understand how these experiences evolve over time in patients and their caregivers. Last, although dyadic interviews provided valuable insights into relational dynamics, the presence of both partners may have constrained the disclosure of more private or conflicting perspectives.

## Conclusion and recommendations

This study offers valuable insights into the experiences of colorectal cancer patients with ostomies and their caregivers, largely aligning with family systems theory while also revealing aspects that extend beyond it, such as cultural factors affecting identity loss, social detachment, and communication barriers regarding body image and intimacy. These findings underscore the necessity for culturally sensitive and relationship-focused interventions to support both patients and caregivers. Future research should explore how to address these specific challenges through a holistic treatment approach that considers emotional and practical needs, emphasizing the importance of gradual social reengagement and open communication within family systems.

## Data Availability

The data will be available from the corresponding author upon reasonable request.
